# Photosynthetic performance of photobionts under intense visible and UV-A radiation in lichens with light-absorbing or light-reflecting fungal pigments

**DOI:** 10.1007/s11120-026-01242-0

**Published:** 2026-10-20

**Authors:** Piotr Osyczka, Natalia Kamińska, Beata Myśliwa-Kurdziel

**Affiliations:** 1 Institute of Botany, Faculty of Biology, Jagiellonian University, Gronostajowa 3, Kraków, 30-387 Poland; 2 Department of Plant Physiology and Biochemistry, Faculty of Biochemistry, Biophysics and Biotechnology, Jagiellonian University, Gronostajowa 7, Kraków, 30- 387 Poland

**Keywords:** Lichen ecophysiology, Photobiont, Light stress, Photosynthesis, Chlorophyll fluorescence, Photosystem II

## Abstract

This study investigated the effect of exposure to direct sunlight and its main components, i.e. visible (VIS) radiation and long-wavelength UV-A (UV-A1; 350–400 nm) radiation, on the photosynthetic performance of two mat-forming epigeic lichens *Cetraria aculeata* (melanic species with light-absorbing cortex) and *Cladonia uncialis* (usnic species with light-reflecting cortex). A short-term (one-hour) irradiation stress was applied to metabolically active thalli and parameters related to photosynthesis at various recovery periods after treatment was examined through complementary in vivo chlorophyll fluorescence techniques (OJIP transients and quenching analysis), 77 K fluorescence emission spectra and assimilation pigment measurements. In both species, considerable but reversible photoinhibition of photosystem II was observed following sunlight and VIS radiation exposure, as evidenced by a significant decrease in F_V_/F_M_ and increased K and J steps of the OJIP curves; albeit photodamage resulting from sunlight was more pronounced. Although UV-A1 had negligible inhibitory effect, it stimulated pigment synthesis in *C. aculeata* and enhanced photochemical energy utilization in *C. uncialis*. The two species revealed different strategies to cope with excess light energy; namely, *C. aculeata* relied on enhanced non-photochemical quenching (NPQ) and redistribution of excitation energy toward PSI, whereas *C. uncialis* preserved a more efficient donor site of PSII (higher F_V_/F_0_). Overall, VIS radiation was identified as the primary factor impairing PSII, posing a substantially greater threat than UV-A1. These findings might be relevant for understanding lichen performance under variable and atypical weather events, such as sunshowers or scattered showers with sunny spells.

**Supplementary Information:**

The online version contains supplementary material available at https://doi.org/10.1007/s11120-026-01242-0.

## Introduction

Lichens are highly specialized symbiotic organisms that exist because of stable association between a fungus (mycobiont) and eukaryotic green algae and/or prokaryotic cyanobacteria (photobiont), further complexed with diverse microbiomes (Lücking et al. 2021; Spribille et al. 2022). Thereby, the lichen combines two entities with different metabolisms and both main partners are involved in the growth of thallus and its performance in the environment. Basically, the mycobiont plays the role of the external partner, forming the thallus structure and providing water with mineral salts, while the photobiont is responsible for the production of assimilates through the photosynthesis process (Hawksworth 1988). A large amount of the produced organic nutrients, except for the part necessary to the existence and development of the photobiont, is supplied to the mycobiont (Johansson et al. 2011). However, the contribution of the photobiont to the lichen biomass is generally much smaller than that of the mycobiont (Collins and Farrar 1978), and in addition the photobiont is considered to be more sensitive to stress factors (Bačkor et al. 2007; Bačkor and Fahselt 2008). Hence, optimal photosynthetic productivity and the balanced physiological functioning of both partners are essential for the lichen to survive in a changing environment (see Stanton et al. 2023). Lichens are generally considered to be stress tolerators (Grime 2001), therefore they colonize a variety of ecological environments at all latitudes. In many open areas, such as deserts, steppes, poor or psammophilous grasslands, tundra and the alpine zone, lichens often constitute the dominant vegetation. High insolation is one of the severe abiotic stresses associated with such habitats, and consequently effective adaptations of lichens to high light conditions are crucial to protect photobionts from irreversible photodamage (Beckett et al. 2021).

Solar radiation reaching the Earth’s surface includes visible and photosynthetically active radiation (PAR; 400–700 nm), UV-A radiation (320–400 nm) and, to a small extent, UV-B radiation (280–320 nm) (Vass et al. 2002). All photosynthetic organisms, although they use life-giving solar energy to produce assimilates, are susceptible to its harmful effects when exposed to excessive irradiance (Barták et al. 2003; Demmig-Adams et al. 1990). Absorption of excess light by the photosynthetic apparatus can lead to the formation of reactive oxygen species (ROS). In turn, ROS imbalance induces oxidative stress and photoinhibition. ROS molecules have harmful effects on nucleic acids, lipids and proteins, which may result in irreversible damage and, consequently, death of photobiont cells (Beckett et al. 2021). The ozone layer blocks most UV-B, while UV-A is only weakly affected by it, therefore most of UV-A reaches the ground and its intensity can be up to 100 times greater than UV-B (Vass et al. 2002). Nevertheless, UV-B is highly energetic and induces many physiological processes, including the synthesis of UV-protective lichen pigments (Solhaug et al. 2003). Many important studies have focused on examining the direct impact of UV-B radiation (e.g. Solhaug and Gauslaa 2004; Nybakken et al. 2004), but still less is known about the effects of UV-A radiation (Solhaug et al. 2003), especially as regards the functioning of the photosynthetic system of photobionts. The physiological impact of UV-A is even referred to as a ‘known unknown’ in plant photobiology because it can acts as both a crucial regulatory signal and a potential stressor (Verdaguer et al. 2017). Excessive UV-A stimulation can elicit a variety of physiological impairments and defensive responses, including similar to those triggered by excess light (see Unal et al. 2009). The physiological impact of UV-A radiation is highly wavelength-dependent, with shorter wavelengths being substantially more effective at inducing high stress (Tyystjärvi 2008).

Recent research in plant photobiology distinguish between two functional sub-regions of UV-A radiation based on their perception mechanisms and physiological effect (Rai et al. 2021), namely short-wavelength UV-A (UV-A2, 315–350 nm) and long-wavelength UV-A (UV-A1, 350–400 nm). Elucidating the effects of this specific sub-region is crucial for understanding its distinct physiological role because UV-A1 constitutes a large fraction of the total solar UV photons reaching the Earth’s surface (Sun et al. 2024). Unlike the shorter and more damaging UV-B and UV-A2 wavelengths, UV-A1 has been shown to potentially propel net photosynthesis with measurable quantum yield in various genotypes (Sun et al. 2024). Therefore, focusing on the UV-A1 range allows for an assessment of its specific contribution to lichen photoprotection and energy balance without the masking effects of severe photodamage caused by the more energetic parts of the UV spectrum.

At the photobiont level, the adaptability to high insolation depends mainly on the plasticity of photosynthetic apparatus (i.e. the ability to dynamically adjust excess-energy dissipation mechanisms), while at the mycobiont level, it is determined by the specific structure of the cortex layer and/or the production of special metabolic compounds that screen the photosynthetic partner from excessive light. Dark, light-absorbing pigments (e.g. melanin) present in the cortex layer and pale, light-reflecting crystals of secondary metabolites (e.g. usnic acid) deposited outside the fungal hyphae can effectively limit the doses of light and UV radiation reaching the algal cells (Gauslaa and Solhaug 2001; Solhaug and Gauslaa 2012; Solhaug et al. 2010).

The overall aim of this study was to investigate the functional response of the photosynthetic apparatus in lichen photobionts affected by short-term intense irradiation, with particular emphasis on the dynamics of photoinhibition and subsequent recovery. We focused on two epigeic lichen species with similar geographical and ecological range but engaging different photoprotective mechanisms, i.e. the synthesis of light-absorbing melanin pigments (as in the case of *Cetraria aculeata*) and the synthesis of light-screening usnic acid (present in *Cladonia uncialis*). Metabolically active lichen thalli were exposed to natural sunlight under field conditions and to selected components of solar radiation under controlled laboratory conditions, i.e., visible (VIS) and long-wavelength UV-A radiation (UV-A1). A relatively short exposure period of one hour was applied to mimic the transient episodes of high irradiance that may affect hydrated lichens in nature. Moreover, we assumed that prolonged periods of high radiation under natural conditions are often associated with progressive dehydration and reduced metabolic activity of lichens. Radiation intensities were selected based on field measurements performed during sunny summer conditions at the study site. We hypothesized that a brief episode of high irradiance induces a sustained reduction in photosynthetic performance, whereby the extent of this impairment and the rate of recovery differ between species due to contrasting photoprotective mechanisms. We combined complementary chlorophyll fluorescence techniques, including OJIP transients and quenching analysis, with low-temperature (77 K) fluorescence spectroscopy and photosynthetic pigment analysis to characterize different aspects of PSII performance and recovery. This integrative approach allowed us to identify which components of the photosynthetic apparatus were most affected by acute light stress and to gain insight into the mechanisms underlying species-specific differences in photoprotection and regeneration.

## Materials and methods

### Target species

Two epigeic lichen species were selected for the study, i.e. *Cetraria aculeata* (Schreb.) Fr. and *Cladonia uncialis* (L.) Weber ex F.H.Wigg. These fruticose lichens have branched thalli that form irregular shrubby tufts (Figs S1A and S1B; for detailed description of the species, see Duke and Purvis 2009; Stenroos et al. 2015). The lichens demonstrate high phenotypic plasticity and, depending on the geographical origin or habitat type, various modifications of the morphology and anatomy of their thalli have been reported (Brodo and Ahti 1996; Pérez-Ortega et al. 2012). Both species are widespread and cosmopolitan, they are especially common in the Northern Hemisphere (Fig. S2) and occur from arctic to temperate zones, including the high Arctic and the subalpine to alpine belts of most mountain ranges (see GBIF Secretariat 2026; Košuthová and Šibík 2013). The lichens are associated mainly with exposed habitats, such as open woodlands, psammophilous or sandy grasslands, steppes and maritime dunes (Printzen et al. 2013; Gheza et al. 2016). Such open habitats are particularly exposed to strong sunlight, which at certain times of the year and at high latitudes can act as a long-lasting adverse factor. Both species may co-occur in one cryptogamic community at the same locality.

Despite ecological similarities, the studied lichens differ considerably in terms of thallus color and develop potentially distinct photoprotective mechanisms. Cortex of *C. aculeata* is three-layered with a brown or almost black outer layer, which is reflected in the darkish color of the thallus. *Cladonia uncialis* is light colored (green-yellow tint) and inherently has a fairly high albedo. Specifically, the firm cortex of *C. aculeata* contain the light-absorbing pigment melanin, while the latter lichen produces the light-reflecting usnic acid (Solhaug et al. 2024). Both lichens also differ in terms of their photosynthetic partner; both contain green algae, but *Cetraria* is associated with *Trebouxia*, while *Cladonia* with *Asterochloris* (Pérez-Ortega et al. 2012; Pino-Bodas and Stenroos 2021).

### Sampling and handling

Lichen material of both species was collected during a rainless period in November 2023 from semi-natural psammophilous grassland located in the vicinity of the Pustynia Błędowska desert (southern Poland). According to the updated Köppen-Geiger climate classification, this region has been included into Cfb type (see Kottek et al. 2006). After being transported to the lab, the thalli were carefully cleaned of soil, plant and bryophytes debris. The material dried at room conditions was frozen (-24 °C) until the analysis. The examined samples of *C. uncialis* were chemically homogeneous (squamatic and usnic acids as the main secondary metabolites), which was confirmed by TLC analysis performed according to standard procedures (Orange et al. 2001). Before the experiments, the thalli were rehydrated (using rainwater equivalent: pH 7.1, conductivity ≅40 µS cm^− 1^) and kept in moist condition under dim light at room temperature (18–20 °C) for 24 h to reach an equilibrium state.

### Experimental design

The lichen thalli were exposed to three types of irradiation stress: (1) direct sunlight – full sun exposure in early July before noon, light intensity ≅1800–1900 µmol photons m^− 2^ s^− 1^ (for basic weather conditions during exposure, see Table S1); (2) VIS radiation – exposure to warm white actinic light emitted by non-heat producing white LEDs, light intensity ≅1800 µmol photons m^− 2^ s^− 1^, room temperature (18–20 °C); (3) UV-A radiation – exposure under UV-A bulbs (Sylvania) emitting radiation with a wavelength of max. 366 nm and covering the UV-A1 spectral range (350–400 nm; see Rai et al. 2021); radiation intensity ≅18 W m^− 2^, room temperature (18–20 °C). The spectral characteristics of the both lamps are provided in Fig. S3.

The UV-A1 irradiance of 18 W m^− 2^ (corresponding to 55–60 µmol photons m^− 2^ s^− 1^) was selected based on direct field measurements performed at the study site. A total UV-A irradiance of approximately 20 W m^− 2^ was recorded simultaneously with a PAR level of about 1800 µmol photons m^− 2^ s^− 1^. The comparable energy flux was reproduced in laboratory conditions under the control of the same measuring devices, thereby the treatment corresponded to the level of UV-A radiation experienced by the thalli in natural conditions, while ensuring stability and repeatability. The thalli were kept in a hydrated state during the irradiation experiments by regular and gentle spraying them with a water mist. The exposure period was always 1 h followed by a recovery; the recovery time intervals were 0.5-h, 2-h, 6-h and 18-h. For the recovery period, samples were placed into a ventilated chamber providing very high humidity (close to 100%) and when moved to a room with dim light (≅10–15 µmol photons m^− 2^ s^− 1^). During the last 15 min of the recovery period, the samples were dark-adapted for in vivo chlorophyll *a* fluorescence measurements. Light intensity (PAR) and UV-A radiation were verified using LI-189 quantum meter equipped with an LI-190 quantum sensor (LI-COR, USA) and Senseca Delta OHM HD 2102.2 photoradiometer equipped with LP471UVA radiometric probe (Senseca, Italy), respectively. The surface temperature of the thalli was monitored throughout the exposure to sun using infrared thermometer (Voltcraft, Switzerland; see Table S1).

### Fast chlorophyll *a* fluorescence induction curves

The photosynthetic activity of PSII was measured in lab conditions based on chlorophyll *a* fluorescence using an advanced continuous excitation chlorophyll fluorimeter Handy PEA+ (Hansatech Instruments Ltd, Norfolk, England). The samples were inserted into leafclips with 4 mm diameter measuring aperture and adapted to darkness for 15 min prior to measurements. The chlorophyll *a* fluorescence transients were induced by ultra-bright red-light (650 nm) provided by an array of three high-intensity LEDs. Typically, a light pulse (1 s) with an intensity of 2500 µmol photons m^− 2^ s^− 1^ was applied; the PEA gain was set to 1.0. The analysis of fast fluorescence kinetics (OJIP) was performed and selected key fluorescence parameters, namely F_V_/F_M_, F_V_/F_0_, ABS/RC, DI_0_/RC, ET_0_/RC, φE_0_ and PI_ABS_ were analyzed (for parameter specifications, see Strasser et al. 2004; Bussotti et al. 2010; Paoli et al. 2010; Kalaji et al. 2016). The measured OJIP curves (averaged data points; *n* = 10) were normalized according to the formula: Vt = (F_t_ − F_0_) / (F_M_ − F_0_) (see Zivcak et al. 2015). Additionally, fluorescence emission was recorded using 1 s light pulses with increasing intensities from 500 to 3480 µmol photons m^− 2^ s^− 1^ at 500 µmol photons m^− 2^ s^− 1^ intervals; the PEA gain was set to 1.0. This was aimed at estimating the light pulse intensity needed to achieve the maximal quantum yield of PSII. In this way, the pattern of chlorophyll fluorescence increase (values of F_0_ and F_M_) in relation to the light pulse intensity was determined for both lichen species.

### Pulse-amplitude modulated chlorophyll fluorescence imaging

Measurements were performed using an Open FluorCam FC 800-O/1010 fluorimeter and FluorCam7 software (PSI, Drasov, Czech Republic) after 0.5-h, 6-h and 18-h of recovery following VIS and UV-A1 radiation. Fully hydrated lichen thalli were placed on round Petri dishes, sprayed with water, covered with lids to prevent water evaporation, and pre-darkened for 15 min before measurements.

Standard protocols provided by the FluorCam7 software were used for *Rapid Light Curve* and *Quenching* measurements, depending on the purpose and specifics of each experiment. All measurements were performed using white actinic light, with the shutter set to 0 and optimal sensitivity (35–40%). The saturating pulse intensity was 3000 µmol photons m^− 2^ s^− 1^. For *Rapid Light Curve measurements*, the actinic light intensity was changed after each saturating pulse, ranging from 29 to 2110 µmol photons m^− 2^ s^− 1^; the curve was collected for 7 light intensity levels, each lasting 60 s. The *Quenching protocol* was modified as described in Osyczka and Myśliwa-Kurdziel (2023). Measurements started from a saturating light pulse (3000 µmol photons m^− 2^ s^− 1^) followed by 1-min dark period. Then, the samples were exposed to white actinic light (200 µmol photons m^− 2^ s^− 1^) for 8 min, followed by 100 s in the dark. Saturating pulses were applied at the end of the actinic light interval and at the end of final dark interval.

Photosynthetic efficiency parameters (QY_max_ and QY) and non-photochemical quenching (NPQ) were calculated using FluorCam7 software. Based on QY, the relative electron transport rate (rETR) was calculated using the formula: rETR = 0.5 × PAR × QY, where QY is the effective PSII quantum yield, PAR is photosynthetically active radiation and 0.5 assumes equal distribution of excitation energy between PSI and PSII (Genty et al. 1989; Björkman and Demmig 1987; Schreiber et al. 1995). The absorbance term (0.84), commonly assumed for leaves of vascular plants (Björkman and Demmig 1987), was intentionally not included, because the fraction of incident light absorbed by the photobiont cells is unknown and varies among lichens because of differences in thallus morphology and cortical pigmentation. Therefore, rETR values calculated according to this approach should be interpreted as comparative rather than absolute values (Green et al. 1998; Solhaug et al. 2021).

### Fluorescence emission spectra at 77 K

Fluorescence emission spectra at 77 K were measured using a Perkin-Elmer LS50B (Perkin Elmer, UK) fluorimeter equipped with a low-temperature module. After exposure to VIS or UV-A1 radiation under laboratory conditions, intact elongated fragments of thalli were promptly inserted into sample tubes (7 cm × 0.3 cm) and immediately frozen in liquid nitrogen, without any dark recovery period prior to freezing. The samples were excited at 440 nm and spectra were recorded in the range of 630–800 nm. Excitation and emission slits were set to 8 and 5 nm, respectively, and the scanning speed was 300 nm min^− 1^. An internal cut-off filter (transmitting wavelengths above 515 nm) was placed in the emission channel. Ten spectra were recorded from five thallus fragments for each species. Between measurements, the tubes were slightly rotated and moved vertically within the liquid nitrogen-cooled sample holder to reduce positional bias. The resulting 50 spectra were corrected for scattering and wavelength-dependent photomultiplier response, then averaged and normalized to equal area.

### Photosynthetic pigments analysis

Immediately after exposure of the thalli to VIS or UV-A1 radiation under laboratory conditions, samples were promptly dehydrated by removing surface water with filter paper and placed in sealed containers filled with silica gel. Dry lichen material (ca. 35 mg) was then carefully washed with CaCO_3_-saturated 100% acetone. The washing procedure consisted of six 1-min rinses with 2 ml of bathing medium with gentle shaking. This procedure was intended to remove compounds capable of chlorophyll degradation during extraction as well as substances potentially interfering with chlorophyll determination (see Barnes et al. 1992). Pigments were extracted twice with 3 ml of dimethyl sulfoxide (DMSO) containing 2.5 mg/ml polyvinylpolypyrrolidone (PVPP) for 45 min at 65 °C in darkness, with shaking at regular intervals at 300 rpm (ThermoMixer C, Eppendorf, Germany). After cooling, the combined extracts (6 ml) were centrifuged for 10 min at 3000 rpm (Sigma 3-18KS, Osterode, Germany). The supernatant was then diluted 1:1 (v/v) with fresh DMSO. Absorbance was measured at 665.1, 649.1, 480, 435, and 415 nm using a UV-VIS spectrophotometer (Jasco V-650, JascoCo, Japan), and concentrations of chlorophyll *a*, chlorophyll *b*, and total carotenoids were calculated according to Wellburn’s equations (Wellburn 1994). The pheophytinisation quotient was additionally determined (Ronen and Galun 1984). Pigment concentrations were expressed on a dry weight basis (mg g⁻¹ DW).To minimize chlorophyll degradation, all steps were performed under dim light conditions.

### Data processing

Since selected fluorescence parameters characterizing PSII functionality were measured on different scales, the values were converted to the range of 0 to 1 in relation to the respective values of control samples (control value is equal to 1; *n* = 10). The statistical significance of differences was assessed using one-way ANOVA (*p* < 0.05) or two-way ANOVA (recovery period after irradiation stress ⋅ lichen species; *p* < 0.05). The Tukey HSD or Games-Howell post-hoc tests were also used for pairwise comparisons.

## Results

### Photosynthetic activity under different light intensities

#### Chlorophyll *a* fluorescence in relation to the light pulse intensity

**Fig. 1:**
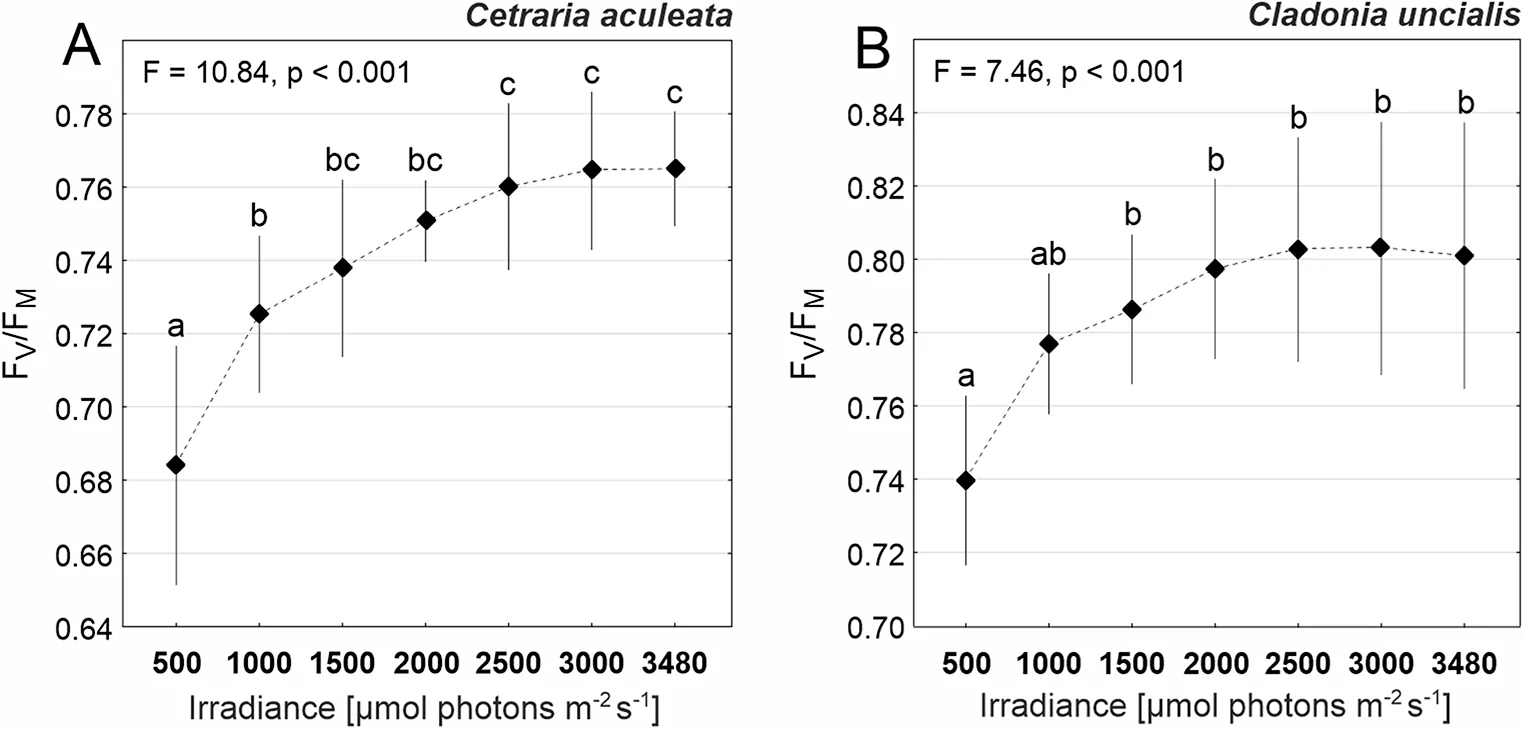
The values (mean ± SD, *n* = 10) of F_V_/F_M_ in relation to the light pulse intensity for dark- adapted lichen samples of *Cetraria aculeata* (**A**) and *Cladonia uncialis* (**B**). The results of one-way ANOVA (F and p values) and Tukey’s (HSD) post-hoc test are provided, various letters indicate statistically significant differences (*p* < 0.05)

Fluorescence signals obtained using the light-pulse method during repeated measurements at increasing pulse intensities (from 500 to 3480 µmol photons m^− 2^ s^− 1^) showed always higher F_V_/F_M_ values for *C. uncialis* than for *C. aculeata* (Fig. 1A and B). For *C. uncialis*, a lower light pulse intensity (around 1000 µmol photons m^− 2^ s^− 1^) was sufficient to approach the maximal quantum yield of PSII, whereas *C. aculeata* required a higher intensity of about 1500 µmol photons m^− 2^ s^− 1^ (compare Fig. 1A and B). Beyond these threshold levels, F_V_/F_M_ continued to increase with increasing light pulse intensity in both species, but these changes were not statistically significant.

#### Rapid light curves

**Fig. 2:**
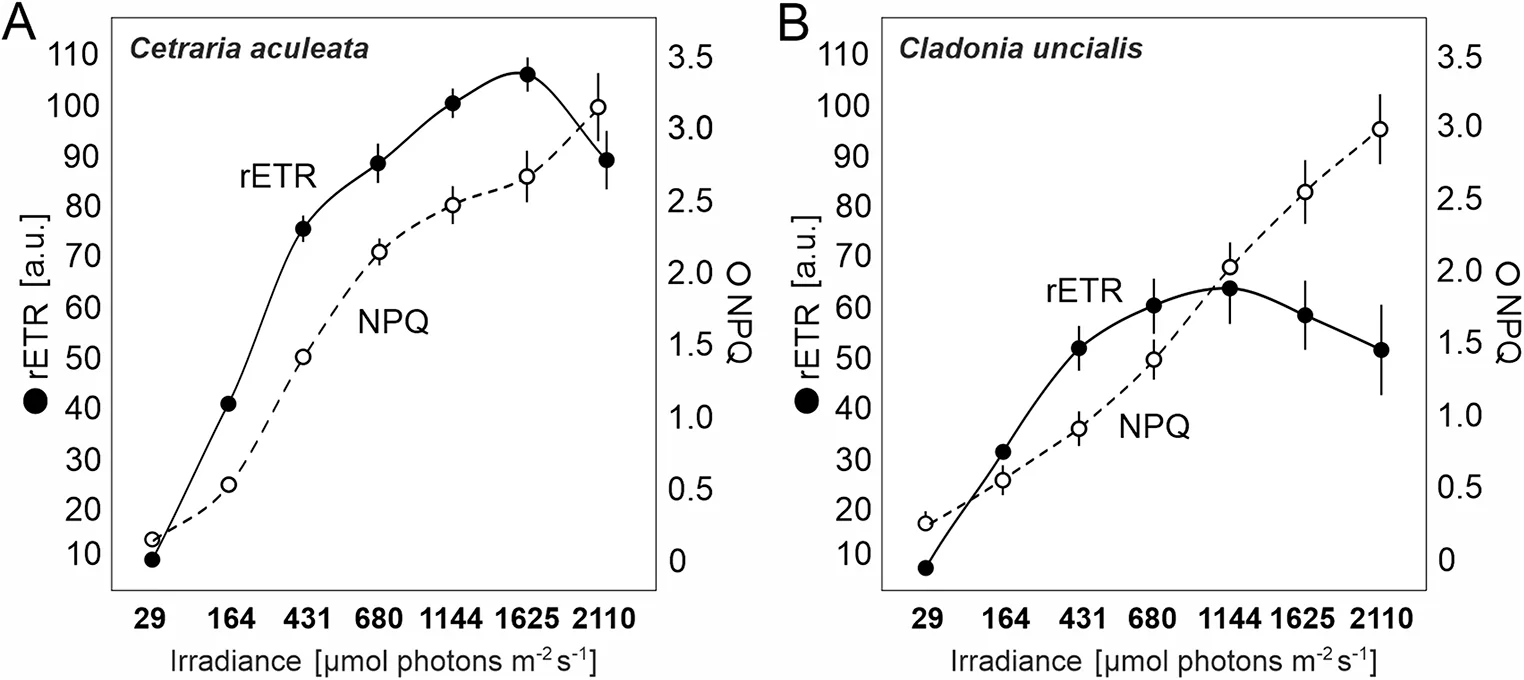
Mean values (*n* = 10) of fluorescence parameters rETR (relative electron transport rate) and NPQ (non-photochemical quenching) for *Cetraria aculeata* (**A**) and *Cladonia uncialis* (**B**) calculated from the fluorescence curves obtained for white actinic light (29–2110 µmol photons m^− 2^ s^− 1^). Error bars show the standard deviation when larger than symbol size

Rapid light curves (RLCs) were measured to assess changes in chlorophyll fluorescence parameters associated with photosynthetic and photoprotective processes, such as relative electron transport rate (rETR) and non-photochemical quenching (NPQ) under increasing light intensities (Fig. 2). rETR values were higher for *C. aculeata* compared to *C. uncialis* across the entire range of light intensities. In both species, a rapid increase in rETR was observed until a maximum value was reached, followed by a decline. The rETR maximum for *C. aculeata* occurred at approximately 1600 µmol photons m⁻² s⁻¹, while for *C. uncialis* at about 1000 µmol photons m⁻² s⁻¹. For both lichens, NPQ values increased rapidly (about 10 times) across the tested light intensity range and reached values up to 3.0. Notably, NPQ values were consistently higher for *C. aculeata* than for *C. uncialis* (compare Fig. 2A and B).

### Recovery kinetics after radiation treatment

#### Normalized OJIP transients

**Fig. 3:**
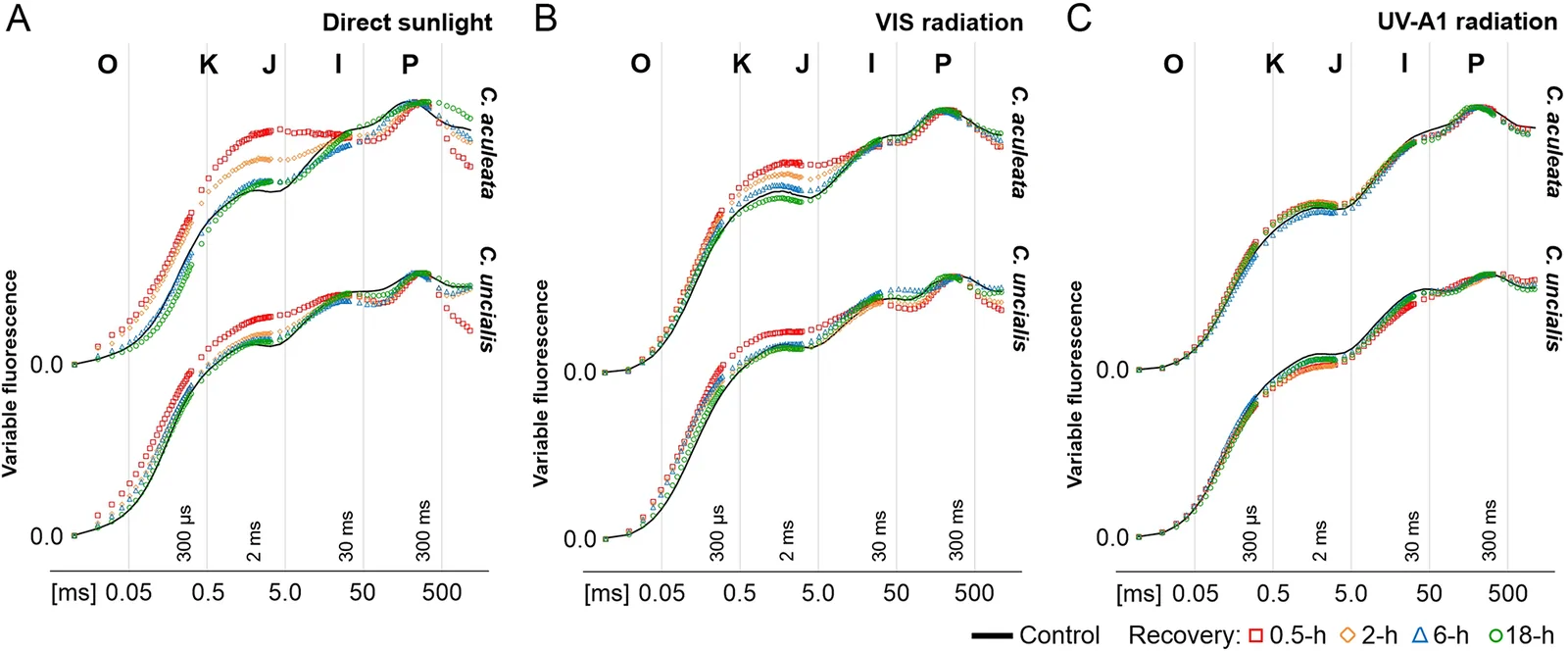
Normalized OKJIP fluorescence transients (see Materials and methods *“Fast chlorophyll fluorescence kinetics”*) for *Cetraria aculeata* and *Cladonia uncialis* after 0.5-h, 2-h, 6-h and 18-h recovery following one-hour exposure to direct sunlight (**A**), VIS radiation (**B**) and UV-A1 radiation (**C**). The curves are plotted based on data points averaged for 10 replicates

Normalized chlorophyll *a* fluorescence induction curves (OJIP) were overlaid to monitor changes in PSII functioning during recovery after short-term exposure to intense light (natural or artificial) or UV-A1 radiation. Regardless of the stress factor, the curves for all samples of both species reveal the characteristic sequence of OKJIP steps. However, the curves obtained after exposure to sun and intense VIS radiation differ from those of the control samples in their general shape, with a stronger distortion effect observed for direct sunlight exposure (Fig. 3A and B). The main difference concerns the K and J steps, which are significantly elevated in these samples. Moreover, the J-I phase became flattened and the intermediate I step was not clearly accentuated. In this respect, the courses of the chlorophyll induction curves were altered to a greater extent for *C. aculeata* than for *C. uncialis*, especially with a view to insolation factor. Irrespective of the degree of curvature change due to stress, the induction curves gradually regain a close to control shape with recovery period. In contrast to excessive VIS, UV-A1 radiation did not result in substantial changes in the shape of the curves (Fig. 3C).

#### JIP test parameters

**Fig. 4:**
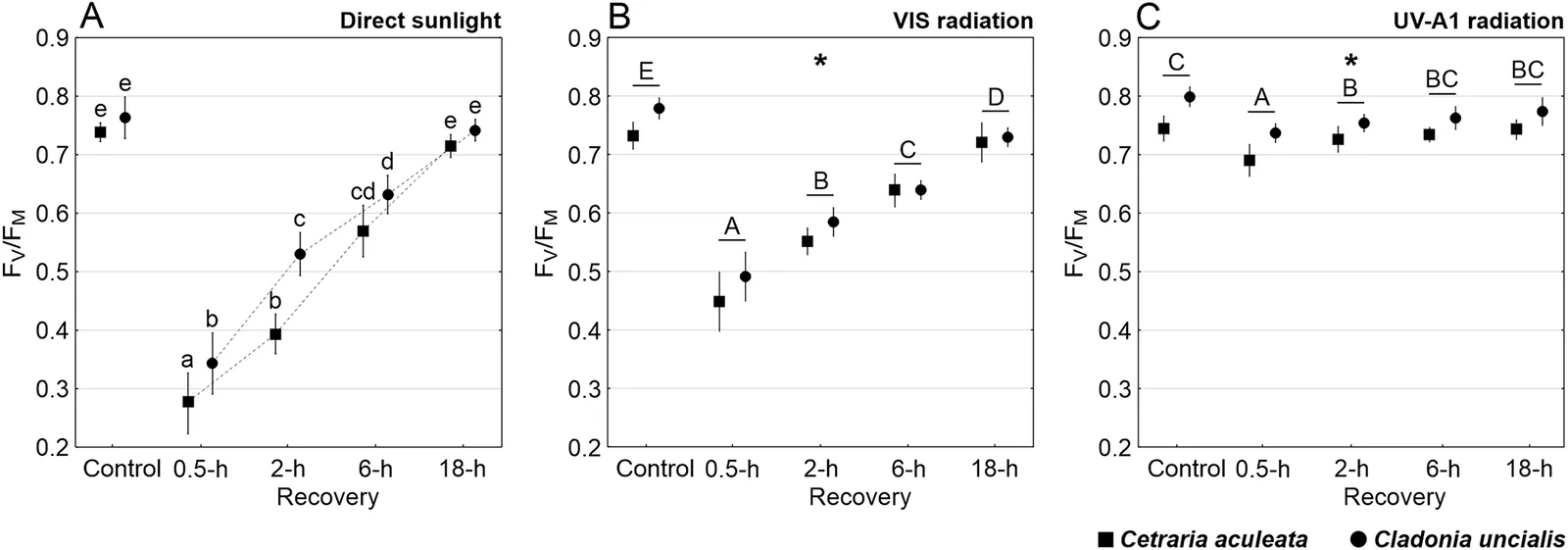
Maximal quantum yield of PSII (F_V_/F_M_ values; mean ± SD, *n* = 10) determined for *Cetraria aculeata* (black squares) and *Cladonia uncialis* (black dots) after 0.5-h, 2-h, 6-h and 18-h recovery following one-hour exposure to direct sunlight (**A**), VIS radiation (**B**) and UV-A1 radiation (**C**). The different letters above the bars indicate statistically significant differences (*p* < 0.05); lowercase letters indicate a statistically significant interaction between the recovery period and lichen species, capital letters indicate a significant effect of the recovery period. The asterisks indicates a statistically significant effect of lichen species. For details on the main effects and interactions, see Table S2

Intense light, regardless of species and light source (natural or artificial), strongly affected the parameters related to photosynthetic performance in hydrated and active lichens. The basic parameter characterizing maximal quantum yield of PSII (F_V_/F_M_) measured after 0.5 h of recovery was considerably lower than in the controls for both species. The values of F_V_/F_M_ gradually increased in subsequent recovery time intervals. However, only after 18 h of physiological rest, the parameter levels were comparable to the control ones. Stress associated with sunlight resulted in the strongest effect (Fig. 4A). F_V_/F_M_ values were significantly affected by recovery period and lichen species; there was also significant interaction between these variables (Table S2). The reduction of the maximal quantum yield of PSII caused by exposure to artificial light (VIS), although considerable and hampering the assimilation process, was not as drastic as in the case of direct sunlight (Fig. 4B). In contrast, UV-A1 radiation had a rather negligible effect in this respect (Fig. 4C) except for a slight decrease in F_V_/F_M_ shortly after exposure. Similarly to sunlight, also in the case of artificial radiation sources (VIS and UV-A1), the F_V_/F_M_ value was influenced by recovery period and species, but with the difference that there was no interaction between variables (Table S2).

**Fig. 5:**
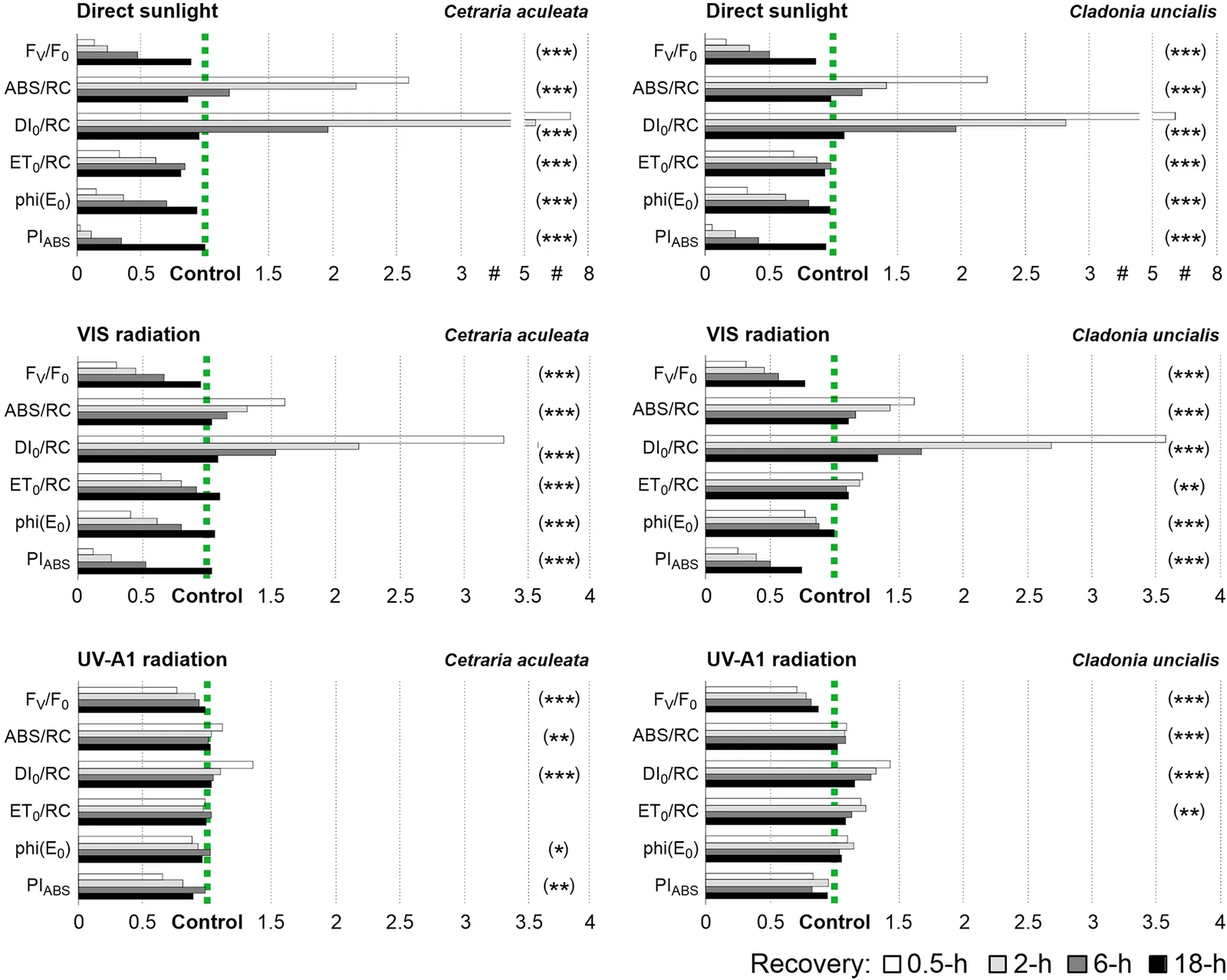
Selected fluorescence parameters characterizing PSII functionality determined for *Cetraria aculeata* and *Cladonia uncialis* after 0.5-h, 2-h, 6-h and 18-h recovery following one-hour exposure to direct sunlight, VIS and UV-A1 radiation. The values are expressed within the range of 0 to 1 in relation to the control value (control value is equal to 1 and is marked with a dashed line; *n* = 10). Asterisks in parentheses indicate significant differences according to one-way ANOVA (*p < 0.05, **p < 0.01, ***p < 0.001); the analysis was performed on raw data

For both species, after 0.5-h recovery from exposure to direct sunlight and VIS radiation, the values of some important fluorescence parameters F_V_/F_0_, ET_0_/RC, phi(E_0_) and PI_ABS_ were markedly lower, whereas ABS/RC and DI_0_/RC were incomparably higher in relation to the control samples. This applies in particular to sunlight stress (Fig. 5). As recovery progressed, the range of differences became smaller and the values of parameters approached those obtained for the control. Relatively small deviations from the control values were observed for UV-A1 radiation (Fig. 5).

#### Chlorophyll *a* fluorescence quenching analysis

**Fig. 6:**
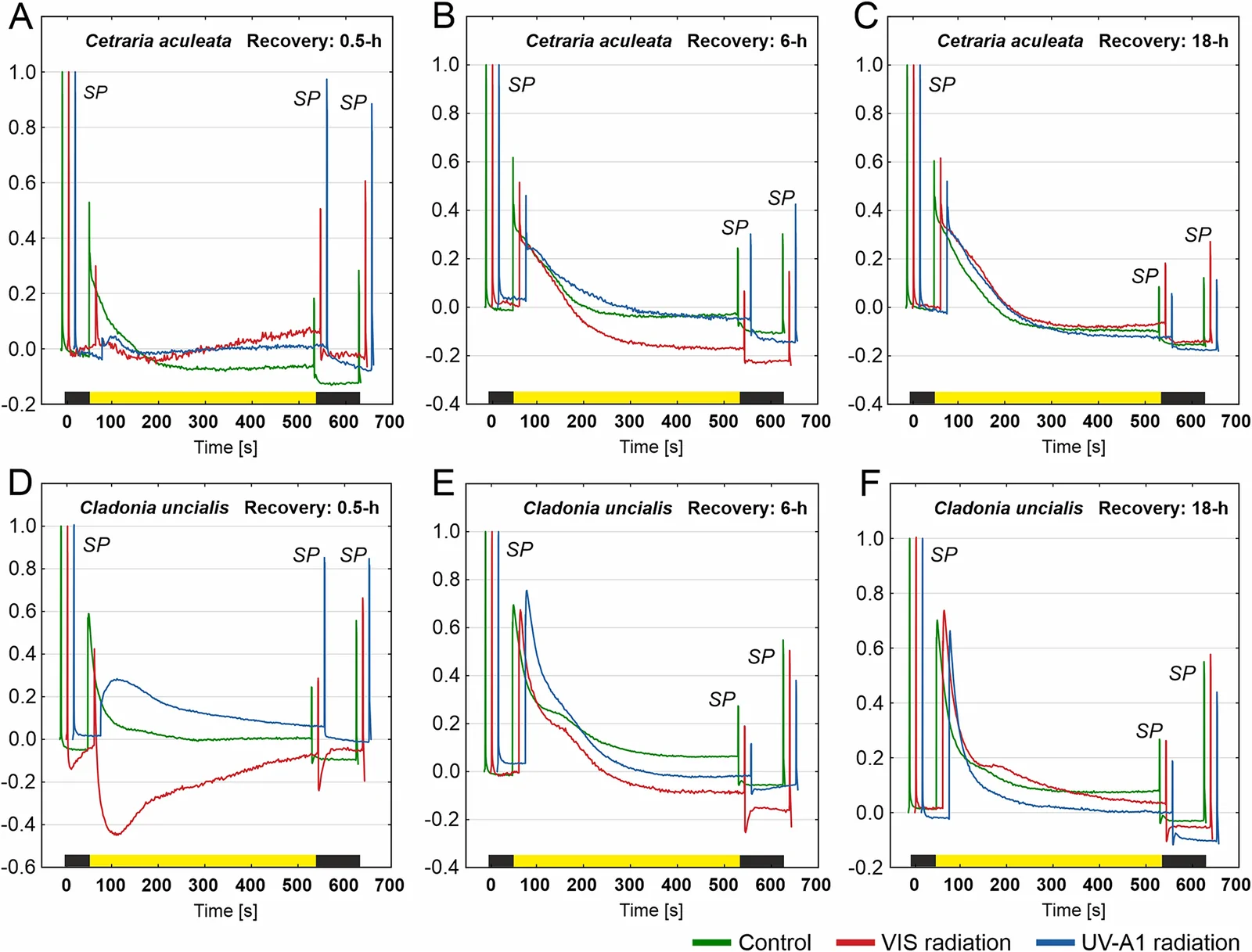
Normalized slow Chl *a* fluorescence induction kinetics determined for *Cetraria aculeata* and *Cladonia uncialis* after 0.5-h (**A, D**), 6-h (**B, E**) and 18-h (**C, F**) recovery following one-hour exposure to VIS and UV-A1 radiation. Black bands indicate dark periods (first – 1 min, last – 100 s), yellow band indicate actinic light period (8 min, 200 µmol photons m^− 2^ s^− 1^); SP – saturating pulse (3000 µmol photons m^− 2^ s^− 1^); the curves are plotted based on an average of 10 repetitions. Quenching curves were normalized such that the F_0_–F_M_ peak values ranged from 0 to 1, which facilitated comparison across samples. Normalization was applied to facilitate comparison across samples (F_0_–F_M_ scaled to 0–1)

In this experiment, slow chlorophyll *a* fluorescence induction curves were measured to assess period after which lichens are able activate photosynthesis under moderate light conditions following intense VIS or UV-A1 radiation. The intensity of the actinic light used in this experiment (200 µmol photons m^− 2^ s^− 1^) was ten times lower than that applied during the stress treatment. Control samples showed a typical fluorescence pattern, generally consistent with the expected responses to saturating pulses and continuous actinic light, as shown in Fig. 6A and F. Moreover, the control curves did not differ considerably between the lichens. Actinic light triggered a rapid increase in the fluorescence signal in both species, with the fluorescence peak (F_p_) remaining below F_M_, although in the case of *C. uncialis* the peak represented a larger fraction of F_M_ when compared to *C. aculeata* (Fig. 6). The pattern of subsequent fluorescence decline under actinic light differentiated the studied lichens: for *C. aculeata*, a sudden decrease was followed by a more progressive decline, whereas in *C. uncialis*, the signal decreased gradually. Moreover, for *C. aculeata*, the fluorescence (F_t_) under actinic light dropped below the initial fluorescence level (F_0_) measured at the beginning of the experiment. After the actinic light phase, a short dark period (100 s) allowed partial recovery of the fluorescence response to a saturating pulse in both species, with recovery being noticeably higher in *C. uncialis*.

The effects of both stimuli, i.e. VIS and UV-A1 radiation, were most pronounced shortly after the treatment. Following VIS exposure, the fluorescence induction curves recorded 0.5 h after the treatment (including 15 min of dark adaptation; Fig. 6A and D) showed an atypical response upon illumination with actinic light of moderate intensity. In both species, turning on the actinic light caused fluorescence decline to the F_0_ level, similar to that typically observed after a saturating light pulse. In *C. aculeata*, fluorescence subsequently reached a local minimum at around 200 s, slightly below the F_0_ level. In *C. uncialis*, the response was even more pronounced, with a drastic decline in fluorescence well below the F₀ level and a gradual return toward this level after approximately 100 s of actinic illumination. After switching off the actinic light, the response to a saturating light pulse was recorded under dark conditions. For both species, the effect of VIS treatment was still visible after 6 and even 18 h of recovery.

For both lichens, 0.5 h after exposure to VIS radiation (including 15 min dark adaptation), QY_max_, QY, NPQ, R_fd_, and F_V_/F_0_ were markedly lower than those of the respective controls (Table S3), whereas qP remained almost unchanged. After 6 h of recovery, all parameters increased, with NPQ and qP returned to the control values; whereas QY_max_, QY and F_V_/F_0_ remained below the controls. After 18 h of recovery, all parameters except R_fd_ and F_V_/F_0_ were similar to their respective controls.

Following UV-A1 exposure, the fluorescence induction curves recorded 0.5 h after treatment differed markedly from those observed after VIS treatment (Fig. 6A and D), particularly directly after the onset of actinic illumination. Unlike the VIS-treated thalli, in UV-A1-treated samples, actinic light caused the characteristic rise in fluorescence signal. For *C. uncialis*, fluorescence rapidly increased to F_p_ level, albeit it was still significantly lower compared to the respective control. After about 40 s of actinic illumination, fluorescence increased further to a maximum value and then gradually decreased. The same general response was observed for *C. aculeata*, although the increase in fluorescence was much less pronounced. In both species, the fluorescence response to a saturating light pulse at the end of the actinic light period was comparable to the response to the first saturating pulse applied to the dark-adapted thalli. In turn, the response of *C. aculeata* to the subsequent saturating pulse, applied after 100 s of darkness, was weaker, which is an atypical phenomenon. Similar to the VIS treatment, the effects of UV-A1 radiation was still observed in both species after 6 and even 18 h of recovery.

In the case of UV-A1 treatment, QYmax remained unchanged in *C. uncialis*, whereas decreased slightly during the recovery of *C. aculeata*. Interestingly, the values of QY and qP were significantly higher for recovery periods of 0.5 h and 6 h compared to the respective controls, particularly for *C. uncialis*. Lowered values of F_V_/F_0_ were observed only in *C. aculeata*. In both species, NPQ and R_fd_ decreased to near-zero levels after 0.5 h of treatment and then gradually increased with the recovery period. After 18 h of recovery, all parameters returned to values comparable to those of the respective controls. Notably, in *C. uncialis*, a slight transient overshoot in NPQ and R_fd_ was observed after 6 h of recovery, with values exceeding those of the control samples.

#### Fluorescence emission spectra at 77 K

**Fig. 7:**
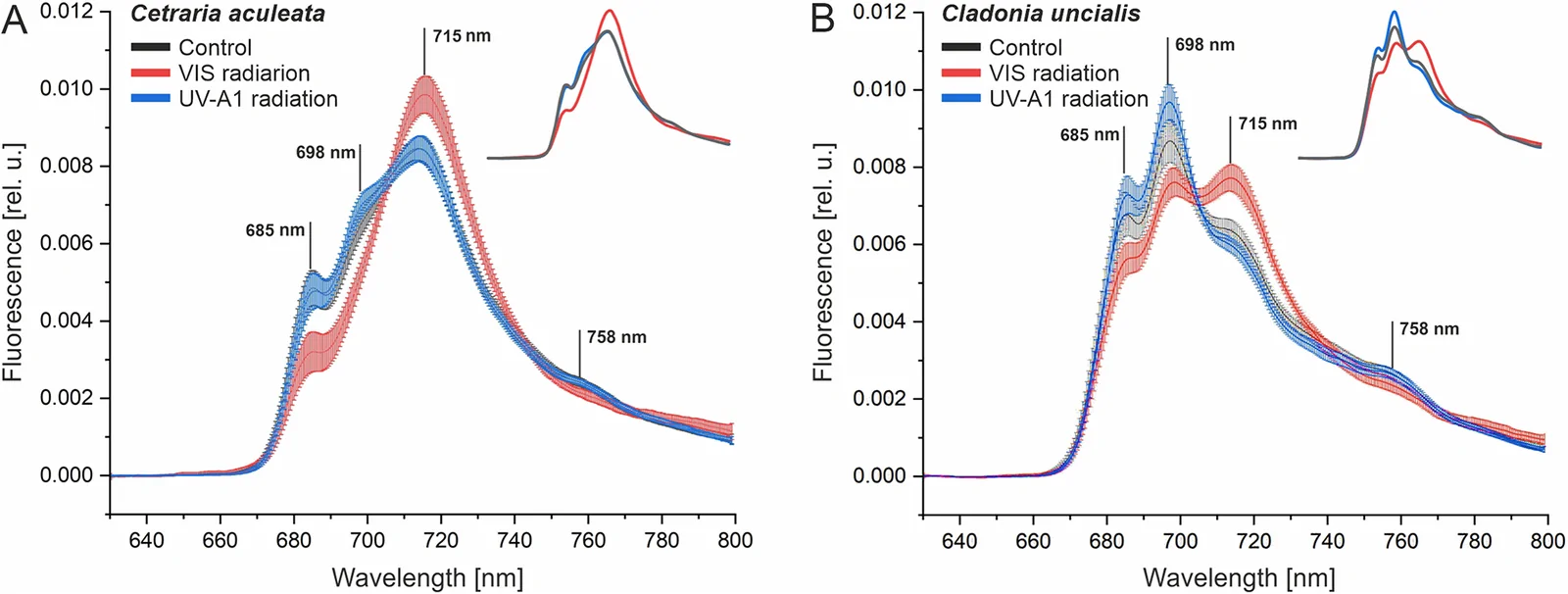
Fluorescence spectra at 77 K (bars indicate SD; *n* = 50) for *Cetraria aculeata* (**A**) and *Cladonia uncialis* (**B**) exposed to one-hour of VIS and UV-A1 radiation. The upper-right insets present the general course of the curves. The spectra were normalized to the area

The fluorescence spectra measured at 77 K for both lichen species were complex and included several overlapping emission bands with maxima at 685, 695–698 and 715 nm. The relative intensities of these bands differed markedly between the two species, and consequently, the overall shapes of their fluorescence spectra were distinct. In the spectrum of *C. aculeata*, the band with a maximum at 715 nm was the most intense and showed only a subtle left shoulder at around 695 nm (Fig. 7A). The band with a maximum at 685 nm was relatively less intense. In contrast, in the fluorescence spectrum of *C. uncialis*, the band with a maximum at 695 nm was the most intense, whereas the 685 nm band demonstrated much lower relative intensity and the 715 nm band was visible only as a subtle right shoulder of the main peak (Fig. 7B).

Exposure to VIS radiation caused distinct changes in the fluorescence spectra. In both species the relative contribution of the 715 nm band increased at the expense of a reduction in the relative intensities of the 685 and 695 nm bands. In *C. aculeata*, the shoulder at around 695–698 nm disappeared after VIS exposure (Fig. 7A). In contrast, for *C. uncialis* the band decreased in intensity at approximately 695 nm but still was observed as a distinct spectral component (Fig. 7B). In turn, after UV-A1 treatment, the fluorescence emission spectra of both lichens remained almost unchanged. It is worth noting that regardless of species, the spectra revealed a very weak band with a maximum in the range of 755–758 nm, which disappeared upon exposure to VIS radiation.

#### Photosynthetic pigments

**Fig. 8:**
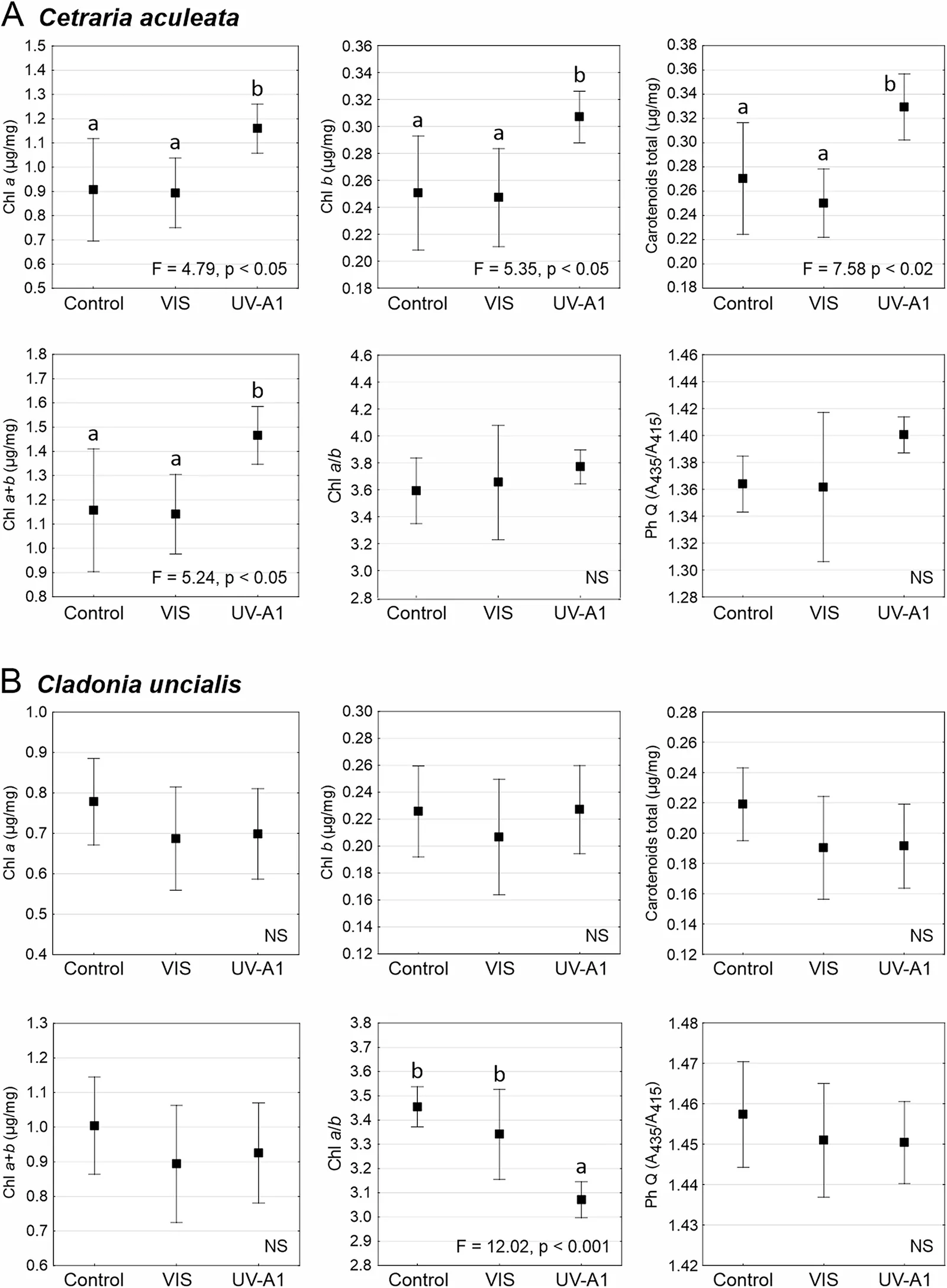
Parameters related to the content of photosynthetic pigments (mean ± SD, *n* = 10) determined for *Cetraria aculeata* (**A**) and *Cladonia uncialis* (**B**) exposed to one-hour of VIS and UV-A1 radiation. The results of one-way ANOVA (F and p values) and Tukey’s (HSD) post-hoc test are provided, various letters indicate statistically significant differences (*p* < 0.05). Ph Q – pheophytinisation quotient, NS – not significant

The referential contents of photosynthetic pigments (and calculated values of related parameters) in the control samples of *C. aculeata* were determined to be slightly higher than in *C. uncialis* (compare Fig. 8A and B); this concerned both chlorophylls and total carotenoids. For the studied lichens, treatment with VIS radiation did not result in any significant changes in pigment contents. Only minor fluctuations with decreasing trends were observed in *C. uncialis* (this also applies to UV-A1 radiation). The most important result concerns the strong effect of UV-A1 on the pigment contents in *C. aculeata*; the lichen responded with increased pigment synthesis. Significantly higher concentrations of chlorophylls and carotenoids were found in the samples after exposure to UV-A1 radiation (Fig. 8A). Roughly, a gain of 20% over the control values was recorded. Although the pigment content in *C. uncialis* was found to be relatively stable, a significant decrease in Chl *a*/*b* upon UV-A1 treatment was noted (Fig. 8B). However, the ratio still remained at a high level.

## Discussion

The common occurrence in a wide geographical range is an attribute of numerous lichens with low habitat requirements or those that tend to thrive in open areas (Liška and Herben 2008). The studied lichens are not highly specialized and can be treated as an epigeic generalist, although they prefer open sites and therefore may be exposed to high temperatures and strong insolation (Jüriado et al. 2016; Ketner-Oostra et al. 2010). Furthermore, both species are widely distributed in the high northern latitudes where daylight may persist for most of the day during the summer season (see Garreau et al. 2026). Unlike forested areas, open landscapes provide dynamic habitats with pronounced fluctuations in local climate conditions within the daily cycle (Chen et al. 1993). In such sites, lichens often have to cope with stress resulting from unstable weather, which is the primary factor determining their hydration status and, consequently, metabolic activity. As demonstrated by Gauslaa and Solhaug (1996), prolonged exposure of air-dry thalli to high light can lead to permanent photoinhibition and chlorophyll bleaching. This is particularly critical because desiccation restricts photosynthetic activity and increases the risk of oxidative damage (Kranner et al. 2003), while repair processes require rehydration for their activation. Moreover, the predicted increase in the frequency and intensity of extreme and unforeseeable weather events (Seneviratne et al. 2021) is likely to further increase environmental pressure on lichens. In this context, sunlight may become an environmental factor that largely determines the success or decline of lichens in exposed habitats. The present study focused on hydrated, metabolically active thalli because only in this physiological state can the primary effects of excess radiation on photosynthesis and subsequent recovery processes be assessed. In particular, PSII repair requires hydrated and metabolically active thalli. Although maximum solar irradiance in nature usually coincides with desiccation, episodes of intense sunlight may also occur when thalli are still hydrated, for example following rainfall or under rapidly changing weather conditions. Moreover, a state of full hydration is not necessary for metabolic activity and it is believed that lichens remain active when their water content does not fall below 40% of the dry weight (Sundberg et al. 1996).

The thallus colors may indirectly indicate their relation with the light factor. In most lichens, the photobionts can only receive light through fungal tissue, and therefore the differential dispersion and/or absorption of light by fungal hyphae inevitably affects both the quality and quantity of light that reaches the algal cells (Rikkinen 1995). Melanic *Cetraria* and usnic *Cladonia* lichens are characterized by clearly contrasting reflectance spectra (see Solhaug et al. 2024). Melanin pigments absorb UV-VIS radiation and dissipate much of the absorbed energy through non-radiative processes associated with the reversible formation of stable free radicals. Hence melanized fungal hyphae of the cortical layer may effectively protect algal cells from the damaging effect of solar radiation (Rikkinen 1995; Gauslaa and Solhaug 2001). In turn, usnic acid (a dibenzofuran derivative) provides photoprotection through strong absorption of UV-A and UV-B radiation (McEvoy et al. 2007). Moreover, its crystals formed in the cortex contribute to scattering of incident visible light. Consequently, increasing concentrations of usnic acid reduce the transmittance of both UV and visible radiation to the photobiont layer. In contrast, *C. aculeata* does not produce aromatic UV-absorbing lichen substances, but only fatty acids (lichesterinic and protolichesterinic), consequently, protection against UV-A1 in this species relies mainly on melanin, which absorbs UV and visible radiation over a broad range of wavelengths. The clear response of *C. aculeata* to UV-A, reflected by an increase in pigment content (Fig. 8A) suggests that melanin screening is incomplete and thus a fraction of UV-A1 radiation reaches the algal layer in this species as well.However, the screening efficiency of the cortex is also influenced by the hydration state of the thallus; hydrated cortices transmit substantially more radiation than air-dry cortices, including in the UV-A range (McEvoy et al. 2007). Therefore, in the present study, performed on hydrated thalli, a fraction of the incident UV-A1 radiation may have reached the photobiont despite the presence of usnic acid. The observed response of the photobionts of *C. uncialis* to UV-A1 further indicates that screening by usnic acid is not always complete. Moreover, the cortex does not constitute a uniform barrier, and local variations in the distribution and concentration of usnic acid may contribute to variation in the amount of UV-A1 reaching the algal layer.

The problem of the adaptive strategy and sensitivity/resistance of the studied lichens to high insolation is more complicated and is not limited only to the photoprotective properties of the fungal pigments and the resulting photosynthetic activity of the algal component. The thalli of the lichens *Cetraria* and *Cladonia* harbor photobionts from different phylogenetic lineages. Although closely related, *Asterochloris* and *Trebouxia* differ in terms of the structure and ontogenetical development of chloroplasts (Škaloud and Peksa 2010; Škaloud et al. 2015). Therefore, differences in the performance of their photosynthetic apparatus cannot be ruled out, for example, due to differences in the organization and/or functional plasticity of photosynthetic antennae.

The rETR light-response curve (Fig. 2) allows for a relative estimate of PSII electron transport rather than the actual electron flow because the absorbance term was not included in the calculation. The light reaching the photobiont layer is attenuated by the upper cortex, which is probably stronger in the melanic *C. aculeata* than in the pale *C. uncialis*. Consequently, the higher rETR for *C. aculeata* across the whole range of irradiance does not necessarily mean a higher real electron transport rate, which depends on the amount of light that actually reaches the photobiont cells, a quantity that is, in fact, difficult to estimate. In addition, the observed decline of rETR at the highest actinic irradiances may be partially due to methodological reasons. The saturating pulse (3000 µmol photons m^− 2^ s^− 1^) was not much stronger than the highest actinic light (2110 µmol photons m^− 2^ s^− 1^), and when strong NPQ is induced the pulse may no longer be fully saturating. This could lead to underestimation of the maximal fluorescence in the light-adapted state (F_M′_) and, consequently, also of QY and rETR. Under dark-adapted conditions, F_V_/F_M_ approached the maximal values at pulse intensities of approximately 1000 µmol photons m^− 2^ s^− 1^ in *C. uncialis* and 1500 µmol photons m^− 2^ s^− 1^ in *C. aculeata.* A slight further increase in F_V_/F_M_ was observed with increasing pulse intensity with a more evident plateau reached at approximately 2500 µmol photons m^− 2^ s^− 1^ (Fig. 1). This suggests that the 3000 µmol photons m^− 2^ s^− 1^ pulse may not have been fully saturating under strong NPQ. Therefore, the decrease in rETR at high irradiance cannot be unequivocally attributed to photoinhibition, particularly given the short duration of the light curve (Fig. 2).Exposure of metabolically active lichen thalli to solar and intense VIS radiation for 1 h induced pronounced alterations in PSII activity, especially shortly after stress (0.5 h of recovery) (Fig. 4; Table S3). The significant decrease in F_V_/F_M_ (Fig. 4) is a well-known indicator of PSII photoinhibition (Björkman and Demmig 1987) reflecting the loss of PSII activity. Recovery from PSII photoinhibition requires repair and replacement of the damaged the D1 protein. The fact that recovery required up to 18 h indicates that the observed impairment was indeed photoinhibition (qI) involving the D1 protein repair cycle rather than transient photoprotective energy dissipation (qE), which relaxes much more rapidly (Tyystjärvi 2008; Nawrocki et al. 2021).

The elevated O-K-J phase (photochemical) of the OJIP curves (Fig. 3; see Lazár 2006 for the review) confirms PSII impairment. The elevated J-step indicates a disturbance in electron transport at the acceptor site (Toth et al. 2007; Zhang et al. 2017) and the increased signal at the K-step points to the damage to the oxygen-evolving complex (OEC) on the donor side of PSII (Strasser et al. 2004). This impairment was more pronounced under the influence of natural sunrays than the intense VIS radiation used in laboratory conditions.

It should be noted, however, that the thalli warm up when exposed to sun and function at a higher temperature regime. On the other hand, the lichens were hydrated throughout the treatment period and the temperature did not approach critical thresholds for their metabolism (Table S1; see Osyczka and Myśliwa-Kurdziel 2024). Nevertheless, the combined effect of high light and elevated temperature likely contributed to the observed damage. In addition, sunlight differed from the artificial VIS source in spectral composition, providing a broader spectrum that included both UV-A1 and UV-A2 radiation (and to some extent UV-B) as well as a relatively stronger blue-light VIS component. Since the efficiency of PSII photoinhibition increases significantly with decreasing wavelength (Tyystjärvi 2008), the presence of these more energetic photons in natural sunlight likely contributed to more severe donor-side photodamage. The manganese cluster in oxygen evolving complex has been identified as one of the primary targets of short-wavelength radiation (Hakala-Yatkin et al. 2010; Hou and Hou 2013). Heat stress may also contribute to PSII inactivation by affecting the oxygen-evolving complex (OEC), which impairs electron transport and increases the K-step amplitude in the OJIP curve (Strasser and Bruno 1997; Srivastava et al. 1997; Oukarroum et al. 2012; Bednařikova et al. 2020).

The JIP-test (Fig. 5) further supported these observations and revealed a significant drop in F_V_/F_0_ indicating a transient limitation on the donor side of PSII, likely due to reduced electron donation from OEC to P680⁺ (Gupta 2020). F_V_/F_0_ also corresponds to the ratio of quantum yield of photochemical to non-photochemical in PSII (Roháček 2002). The increases in ABS/RC and DI_0_/RC, observed in both investigated lichen species, reflect higher relative energy absorption per active reaction centre and greater dissipation of excitation energy in the form of heat or fluorescence, respectively. Concurrently, in the case of *C. aculeata*, ET_0_/RC and φE_0_ decreased, pointing to a limitation on the PSII acceptor side and reflecting restricted electron transport beyond Q_A_ (Tsimilli-Michael 2020). Interestingly, for *C. uncialis* after high light treatment, φE₀ decreased while ET_0_/RC increased. This pattern likely reflects stress-induced adjustment of electron transport efficiency.

A marked reduction in F_V_/F_0_ was also evident in the quenching experiments performed under moderate actinic light (Table S3). This confirms that the functional impairment of PSII persisted during recovery and was observed even under considerably lower actinic light with respect to that applied during the high-light treatment. This persistent drop in efficiency is characteristic of photoinhibition (qI), which requires the D1 protein repair cycle for recovery and is distinct from rapidly reversible protective quenching (Tyystjärvi 2008). In addition, a rapid decrease in fluorescence immediately after the onset of actinic light was observed, especially in the case of *C. uncialis.* Both the effective quantum yield of PSII (QY) and NPQ were reduced compared to the respective control samples. The limited NPQ capacity suggests that a large fraction of PSII reaction centres had already become photoinhibited (qI), thereby reducing the capacity for rapidly reversible non-photochemical quenching (qE). These findings indicate that intense VIS radiation not only transiently impairs electron donation on the donor PSII side but also decreases the number of active PSII reaction centres and limits energy dissipation. Together with the OJIP results (decreased φE_0_ and increased ET_0_/RC in *C. uncialis*), these observations point to partial inactivation of PSII centres, compensatory redistribution of electron flux among remaining functional centres and an overall reduction in steady-state photochemical efficiency. Different responses of *C. uncialis* and *C. aculeata* suggest species-specific strategies to tolerate excess light energy (see also Solhaug et al. 2024), with *C. uncialis* maintaining a more robust water-splitting capacity despite partial inactivation of reaction centre.

Analysis of 77 K chlorophyll fluorescence emission spectra, recorded immediately after VIS treatment, provided a snapshot of the functional state of the photosynthetic apparatus at the end of intense VIS exposure. This helps explain the observed differences between the two studied species. A relative decrease in fluorescence emission at 685 nm and 695–698 nm (Fig. 7), originating predominantly from the PSII core antenna, together with a relative increase in emission at 715 nm, typically associated with PSI (Lamb et al. 2018; Andreeva et al. 2003; Yamamoto et al. 2013), was observed in both species. This suggests either a redistribution of excitation energy toward PSI or accumulation of excitation energy in PSI when PSII photochemistry becomes transiently impaired. These spectral changes are consistent with in vivo chlorophyll *a* fluorescence analyses and support the conclusion that intense VIS radiation induces reversible changes in PSII function in lichen photobionts.

In *C. aculeata*, the increase in relative PSI fluorescence at 77 K was accompanied by a decline in QY_max_ and a marked reduction in non-photochemical quenching (NPQ), which may reflect a partial and compensatory transition to State 2 involving redistribution of excitation energy from PSII to PSI (Ruban and Johnson 2009; Osyczka and Myśliwa-Kurdziel 2023). In contrast, *C. uncialis* exhibited less pronounced changes in both NPQ and the 77 K fluorescence spectra (Table S3 and Fig. 7). This indicates a relatively limited engagement of state transitions and a reduced capacity to relieve excitation pressure on PSII. In this species, the band at ~ 695 nm decreased in intensity but remained clearly detectable after VIS exposure, indicating a different pattern of excitation energy redistribution compared to *C. aculeata*. The JIP test results (decreased φE_0_ and altered ET_0_/RC; Fig. 5) and the pronounced decrease in fluorescence (F_t_ falling well below the F_0_ level after 0.5 h of recovery; Fig. 6d) measured under moderate actinic light indicate species-specific responses to intense VIS radiation, involving partial PSII inactivation, compensatory electron flow and diggerent patterns of non-photochemical energy dissipation (see also Balarinová et al. 2014; Gasulla et al. 2019; Solhaug et al. 2024).

In contrast to VIS radiation, UV-A1 treatment did not induce any detectable changes in the 77 K fluorescence spectra (Fig. 7), suggesting that no substantial redistribution of excitation energy between the photosystems occurred. This is consistent with the relatively slight impairment of PSII function observed under UV-A1. The JIP test, performed after a short recovery period (0.5 h), revealed a slight increase in energy dissipation (DI_0_/RC; Fig. 5) in both lichens, with almost no effect on PSII photochemistry (ET_0_/RC and φE_0_) in *C. aculeata* and with some stimulation in the case of *C. uncialis.* In contrast, the maximal quantum yield of PSII as well as the performance index (PI_ABS_) decreased, especially in *C. aculeata.* At the same recovery period, a notable increase in PSII efficiency (QY) and qP values with strongly reduced NPQ was observed under moderate intensity of actinic light followed UV-A1 treatment and recovery. (Table S3). This suggests that UV-A1 exposure was associated with a transient shift in energy allocation towards photochemical utilization, accompanied by reduced NPQ induction under the applied actinic light condition. This effect is particularly evident in *C. uncialis* where the elevated F_V_/F_0_ ratio compared to the control (Table S3) may indicate changes associated with the donor side of PSII.

Consequently, UV-A1 exposure appears to induce a transient shift in absorbed energy utilization towards photochemistry, which was reversible during recovery. Nevertheless, long-term UV-A1 exposure (24–72 h) can lead to declines in PSII efficiency, chlorophyll degradation, structural alterations in the thallus and even DNA mutations (Unal et al. 2009). In addition, prolonged exposure has been shown to trigger the synthesis of UV-screening pigments, such as parietin and melanins (Solhaug et al. 2003), although UV-B stimulates this process more strongly than UV-A1. The UV-A photoinhibitory effect increased with decreasing wavelength (Tyystjärvi 2008). Accordingly, our conclusions concern the UV-A1 range only at an irradiance of 18 W m^− 2^ and cannot be extrapolated to UV-A2 or UV-B, which are present in sunlight and are more damaging. Remarkably, in the case of *C. aculeata*, we recognized short-term UV-A1 stress as an effective incentive to increased synthesis of assimilation pigments, both chlorophylls and carotenoids (Fig. 8A). It cannot be ruled out, on other hand, that the increase in pigment content (per dry mass of the thallus) was caused by increased division of algae cells inside the thallus in the response to UV-A1 exposure. The photobionts of *C. uncialis* were completely indifferent to this factor in this respect (Fig. 8B). In contrast, one-hour treatment with intense VIS radiation did not cause any significant changes in the content of photosynthetic pigments in both lichens.

## Conclusions

The visible portion of solar radiation was identified as the main contributor to PSII impairment; excessive VIS radiation poses a greater risk of photodamage than UV-A1, whereas UV-A1 appears to play a regulatory role in photosynthetic energy utilization. Short-term exposure of metabolically active lichens to intense VIS radiation or direct sunlight causes PSII photoinhibition in the photobionts of both species, while almost complete recovery required at least 18 h. This photoinhibition was largely reversible and did not indicate permanent structural damage to PSII. Both investigated lichens demonstrate comparable thermal dissipation capacity, suggesting similar photoprotective potential, with NPQ showing no saturation even at increasing light intensities. However, species-specific strategies are evident, including a more robust water-splitting capacity in *C. uncialis* and efficient antenna coupling/PSI activity in *C. aculeata*.

Short-term UV-A1 induced a transient shift in energy utilization towards photochemistry in *C. uncialis*. In contrast, short-term UV-A1 stimulated the accumulation of assimilation pigments, including both chlorophylls and carotenoids, in *C. aculeata*.

Finally, our results suggest that atypical diurnal hydric conditions, such as sunshowers or scattered showers with sunny spells, which compel lichens to enter a metabolically active state during intense solar radiation, may represent a source of physiological stress and potentially limit growth in exposed ecosystems.

## Supplementary Information

Below is the link to the electronic supplementary material.

Supplementary Material 1

## Data Availability

Raw data are available from the authors on reasonable request.
