# Supplementary material for: Photosynthetic performance of photobionts under intense visible and UV-A radiation in lichens with light-absorbing or light-reflecting fungal pigments
Source: Photosynth Res. 2026 Oct 20;164(6):55. doi: 10.1007/s11120-026-01242-0 (PMC13683538; doi:10.1007/s11120-026-01242-0)
Supplement: Supplementary file 1 [file 11120_2026_1242_MOESM1_ESM.pdf]

SUPPLEMENTARY MATERIAL

Photosynthetic performance of photobionts under intense visible and UV-A radiation in lichens with light-absorbing or light-reflecting fungal pigments

Piotr Osyczka[1], Natalia Kamińska[1], Beata Myśliwa-Kurdziel[2]
[1] Institute of Botany, Faculty of Biology, Jagiellonian University, Gronostajowa 3, Kraków 30-387, Poland
[2] Department of Plant Physiology and Biochemistry, Faculty of Biochemistry, Biophysics and Biotechnology, Jagiellonian University, Gronostajowa 7, 30-387 Kraków, Poland

Corresponding author: b.mysliwa-kurdziel@uj.edu.pl

Fig. S1 Epigeic lichens selected for the study: **A** – *Cetraria aculeata* (Schreb.) Fr. (melanic species with dark light-absorbing melanin), **B** – *Cladonia uncialis* (L.) Weber ex F.H. Wigg. (usnic species with pale light-reflecting usnic acid).

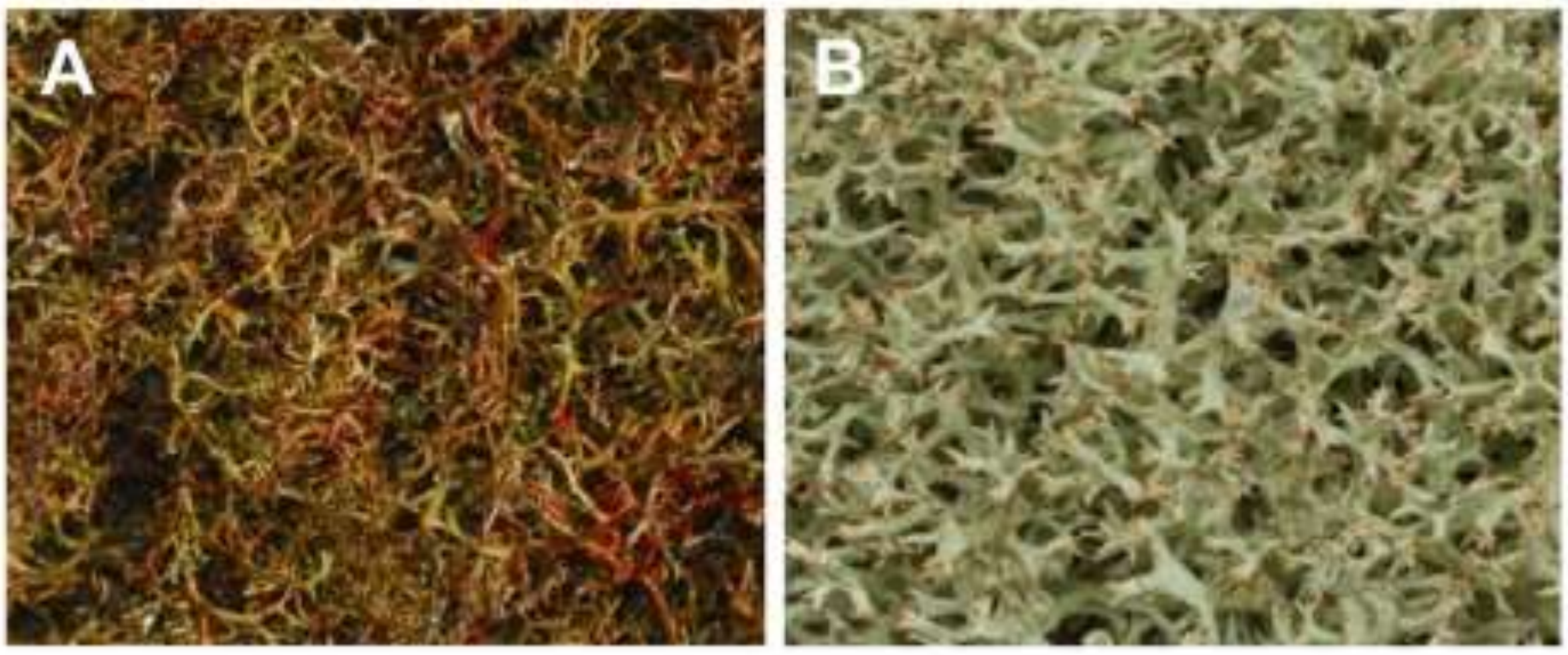

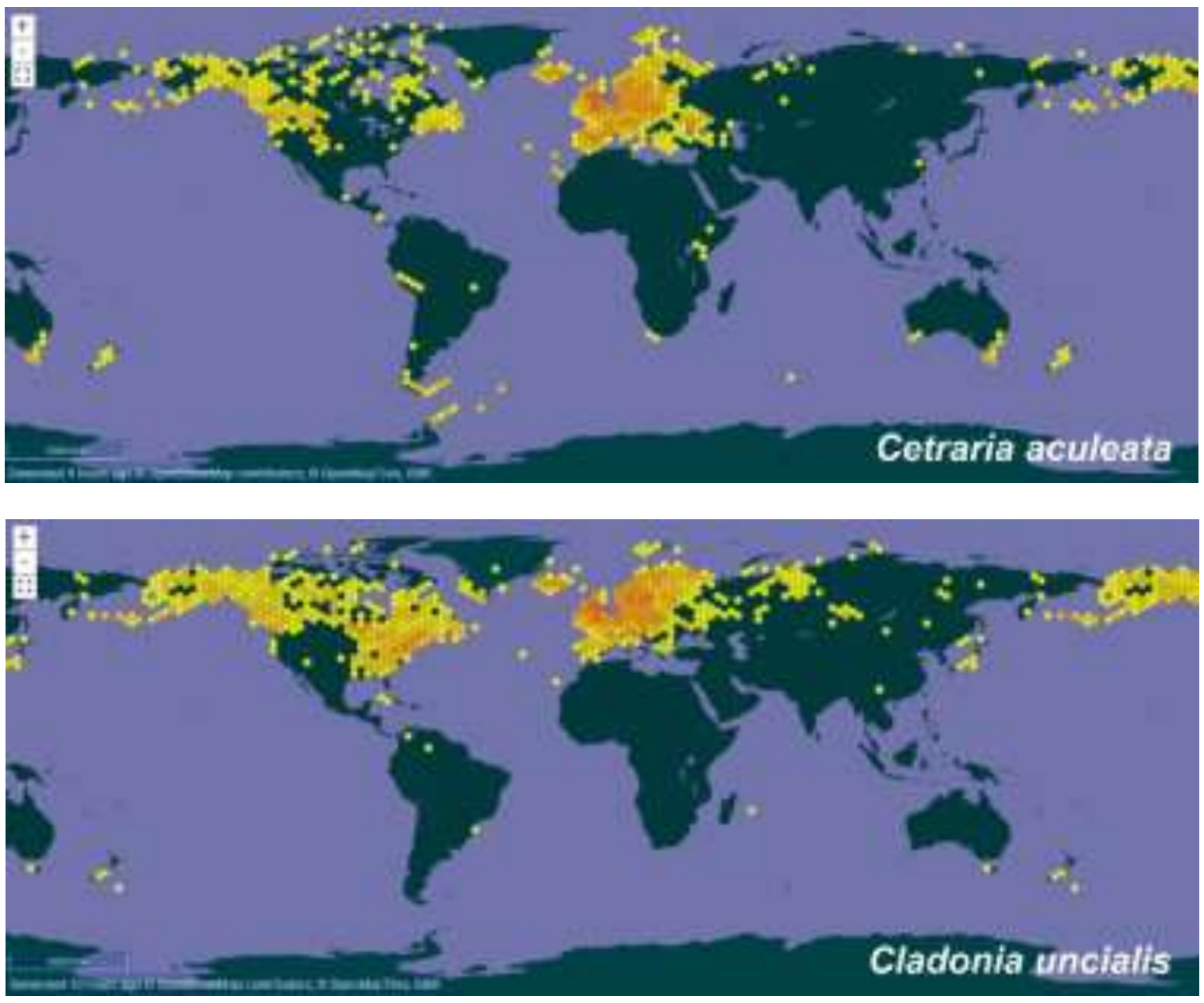

Fig. S2 Global distribution of the studied lichens (based on GBIF – the Global Biodiversity Information Facility: https://www.gbif.org/species/2605275, https://www.gbif.org/species/6606867)

Fig. S3 Emission spectra of artificial radiation sources used in the experiments (measured with an Ocean Optics fiber-optic spectrometer): **A** – UV-A lamp, **B** – white LEDs.

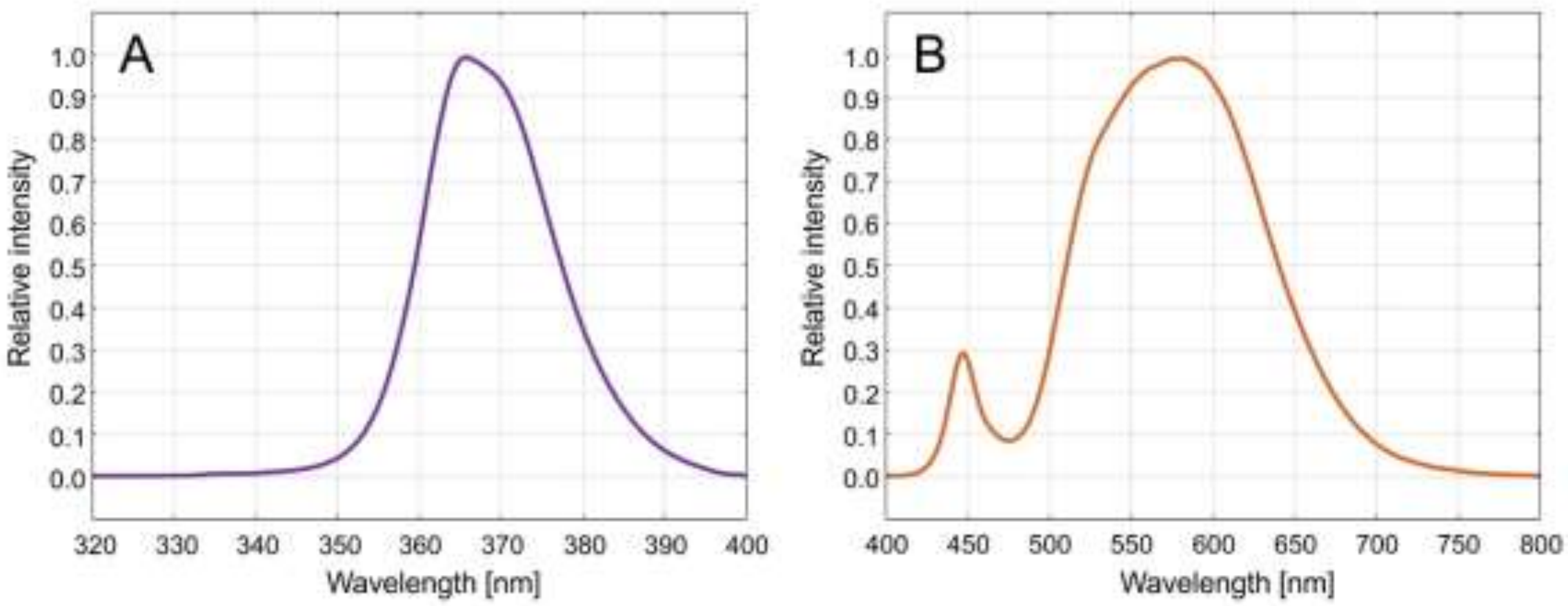

Table S1. Average surface temperature of the examined lichens and basic weather conditions determined during sunlight exposure of the thalli.

	Cetraria aculeata	*Cladonia uncialis*
**Thallus surface temperature** (Infrared Thermometer, Voltcraft)	**31.6±3.4** (mean±SD, n=20)	**28.8±1.6** (mean±SD, n=20)
**Air temperature**	29.3–29.7 °C	
**Relative humidity (RH)**	40–42%	
**PAR** (photosynthetically active radiation; Kipp & Zonen PAR Quantum Sensor)	≅1800–1900 μmol photons m^{-2} s^{-1}	
**Wind speed**	6–10 km/h (data obtained from the nearest meteorological station)	

Table S2. Results of two-way ANOVA ($p < 0.05$) for the effects of recovery period after exposure to irradiation stress (RP; 0.5-h, 2-h, 6-h and 18-h) and lichen species (SP; *Cetraria aculeata* and *Cladonia uncialis*) and their interaction (RT × SP) on the potential quantum yield of PSII (F_V/F_M value). The effects in bold are statistically significant.

Stress	Variables	SS (Sum of Squares)	MS (Mean Square)	DF (Degrees of Freedom)	η^2	F	p
**Direct sunlight**	RP	2.76	0.69	4	0.93	**314.65**	**< 0.001**
	SP	0.09	0.09	1	0.32	**42.84**	**< 0.001**
	RP × SP	0.04	0.01	4	0.18	**4.97**	**0.001**
	Error	0.19	0.002	90			
**VIS radiation**	RP	1.09	0.27	4	0.93	**307.29**	**< 0.001**
	SP	0.02	0.02	1	0.18	**20.05**	**< 0.001**
	RP × SP	0.01	0.002	4	0.09	2.38	0.06
	Error	0.08	0.0009	90			
**UV-A1 radiation**	RP	0.04	0.01	4	0.53	**25.7**	**< 0.001**
	SP	0.03	0.03	1	0.51	**95.7**	**< 0.001**
	RP × SP	0.003	0.0007	4	0.08	2.0	0.11
	Error	0.03	0.0003	90			

Table S3. Selected parameters (mean ± SD, n = 10) calculated from the curves of slow fluorescence kinetics (see Fig. 6 in the main text of the article) determined for *Cetraria aculeata* and *Cladonia uncialis* after 0.5-h, 6-h and 18-h recovery following one-hour exposure to VIS and UV-A1 radiation.

QY_{max} – maximum photochemical efficiency of photosystem II (PSII); corresponds to F_V/F_M
QY – steady-state PSII quantum yield measured in light adapted state
NPQ – steady-state non-photochemical quenching
qP – coefficient of photochemical quenching in the steady-state
Rfd – fluorescence decline ratio in steady-state
F_V/F_0 – ratio of variable fluorescence ($F_V = F_M – F_0$) to fluorescence origin (F_0)

			**QY_{max}**	**QY**	**NPQ**	**qP**	**Rfd**	**F_V/F_0**
Cetraria aculeata	**Control**	**Recovery: 0.5-h**	0.73±0.01	0.45±0.06	1.53±0.48	0.87±0.11	1.93±0.43	2.75±0.16
	**VIS radiation**		0.48±0.05	0.34±0.04	0.21±0.13	0.77±0.04	0.27±0.13	0.94±0.18
	**UV-A1 radiation**		0.64±0.02	0.62±0.03	0.06±0.11	0.99±0.03	0.13±0.11	1.78±0.19
	**Control**	**Recovery: 6-h**	0.75±0.01	0.47±0.02	1.31±0.2	0.84±0.03	1.97±0.27	2.98±0.1
	**VIS radiation**		0.62±0.04	0.36±0.04	1.53±0.74	0.92±0.09	1.68±0.63	1.62±0.28
	**UV-A1 radiation**		0.70±0.02	0.48±0.03	1.06±0.21	0.90±0.04	1.36±0.12	2.36±0.17
	**Control**	**Recovery: 18-h**	0.74±0.01	0.44±0.02	2.21±0.59	0.93±0.1	2.82±0.57	2.87±0.11
	**VIS radiation**		0.69±0.01	0.4±0.02	1.32±0.24	0.82±0.03	1.68±0.23	2.17±0.12
	**UV-A1 radiation**		0.72±0.01	0.41±0.02	2.29±0.56	0.92±0.08	2.45±0.6	2.57±0.13
Cladonia uncialis	**Control**	**Recovery: 0.5-h**	0.73±0.01	0.39±0.03	1.27±0.29	0.72±0.04	1.65±0.35	2.66±0.14
	**VIS radiation**		0.47±0.05	0.27±0.03	0.51±0.14	0.73±0.04	0.49±0.17	0.89±0.16
	**UV-A1 radiation**		0.76±0.02	0.69±0.03	0.15±0.03	0.94±0.02	0.44±0.08	3.15±0.33
	**Control**	**Recovery: 6-h**	0.78±0.01	0.38±0.03	1.38±0.4	0.63±0.03	1.90±0.4	3.58±0.15
	**VIS radiation**		0.55±0.04	0.28±0.03	0.84±0.23	0.70±0.04	1.10±0.26	1.23±0.19
	**UV-A1 radiation**		0.76±0.02	0.33±0.03	2.19±0.4	0.65±0.04	2.88±0.47	3.18±0.29
	**Control**	**Recovery: 18-h**	0.80±0.01	0.37±0.04	1.54±0.51	0.6±0.04	2.01±0.5	3.94±0.16
	**VIS radiation**		0.70±0.03	0.33±0.02	1.13±0.39	0.64±0.05	1.58±0.45	2.31±0.29
	**UV-A1 radiation**		0.75±0.02	0.37±0.02	1.70±0.48	0.69±0.05	2.14±0.52	3.02±0.26
